# The effect of pegbovigrastim on circulating neutrophil count in dairy cattle: A randomized controlled trial

**DOI:** 10.1371/journal.pone.0198701

**Published:** 2018-06-28

**Authors:** Sabrina J. Van Schyndel, Jérôme Carrier, Osvaldo Bogado Pascottini, Stephen J. LeBlanc

**Affiliations:** 1 Department of Population Medicine, Ontario Veterinary College, University of Guelph, Guelph, Ontario, Canada; 2 Elanco Canada Limited, Guelph, Ontario, Canada; University of Illinois, UNITED STATES

## Abstract

Previous research in various species has shown that granulocyte-colony stimulating factor stimulates the production and release of neutrophils from bone marrow. The objective of this study was to characterize the effects of polyethylene glycol-bound bovine granulocyte colony-stimulating factor (pegbovigrastim; Imrestor, Elanco) on circulating leukocyte counts. Thirty-four Holstein cows were randomly assigned to receive 2 injections of either physiologic saline (*n* = 16) or pegbovigrastim (*n* = 18), 7 days before expected calving (d -7) and within 24 hours after calving (d 0). Cows were sampled at d -7, d -6, d 0, d +1, d +7, and d +21, relative to calving. Only cows for which the interval from the first injection to calving was ≥ 4 d and ≤ 10 d were included, such that the interval (mean ± SD) from first treatment to calving was 6.7 ± 1.9 d. Treatment effects were assessed with mixed linear regression models. After the first injection, neutrophil counts (×10^9^/ L) in pegbovigrastim-treated cows increased from 4.3 (95% CI 3.8 to 4.8) at d -7 to 18.2 (CI 16.3 to 20.3) at d -6 (*P* < 0.0001). Their counts then decreased from d -6 to d 0, when the second injection was administered, at a rate of -0.31 ×10^9^ neutrophils/L/day (*P* < 0.0001). After the second injection, neutrophil counts increased from 16.4 (CI 13.7 to 19.6) at d 0 to 32.8 (CI 25.2 to 42.7) at d +1 (*P* < 0.0001), after which counts decreased at a rate of -3.73 ×10^9^ neutrophils/L/day until d +7 (*P* < 0.0001). Counts continued to decrease from d +7 to d +21 at a slower rate of -0.43 ×10^9^ neutrophils/L/day (*P* < 0.0001), until baseline levels were reached. Conversely, in control cows, neutrophil counts were unchanged from d -7 to d -6 (*P* = 0.86) after the first injection and then decreased from 6.1 (CI 5.0–7.3) at d 0, to 3.2 (CI 2.4–4.2) at d +1 (*P* < 0.0001) after the second injection. Neutrophil count was greater (*P* < 0.001) in pegbovigrastim-treated than in control cows at days -6, 0, +1 and +7. Area under the curve (cells ×10^9^/ L per 28 d) for neutrophil counts in the pegbovigrastim group was 429, versus 99 in the control group (*P* < 0.0001). The response to each injection of pegbovigrastim was additive and consisted of 95% segmented neutrophils, suggesting that the effect of the treatment was to release mature neutrophils from a substantial pool available in the bone marrow. The sustained increase in circulating neutrophil count around the time of calving may contribute to improved health during the peripartum transition period.

## Introduction

During the periparturient period, dairy cows are at increased risk of metabolic disorders and infectious disease. Diseases in early lactation, such as mastitis, retained placenta, and reproductive tract diseases can lead to high treatment costs, decreased milk production, and impaired reproductive performance [1]. The well-documented negative energy balance (NEB) experienced by dairy cows in early lactation contributes to a reduction in immune function and a predisposition to disease. Uterine disorders and mastitis are associated with decreased neutrophil and lymphocyte function, which are in turn associated with decreased dry matter intake (DMI) and a greater degree of NEB [2, 3]. Infection and inflammation trigger cytokines and chemokines to direct the production and release of leukocytes from bone marrow and their migration to the site of infection. Granulocyte colony-stimulating factor (G-CSF) is a growth factor that stimulates the differentiation of hematopoietic stem cells to granulocytes [4]. Differentiation and maturation from a myeloblast in the bone marrow to a neutrophil in circulation is driven primarily by G-CSF [4]. Injection of G-CSF stimulates proliferation and increases the number of mature neutrophils in circulation. Daily injections of recombinant bovine G-CSF have been shown to significantly reduce immune suppression and neutropenia in dairy cattle [5]. Covalently binding polyethylene glycol (PEG) to the recombinant bovine G-CSF protein (PEG-rbG-CSF) produced increased numbers of neutrophils in circulation with increased myeloperoxidase production for 10 to 14 days after injection [6]. Previous studies exploring pegbovigrastim in dairy cows demonstrated increases in circulating neutrophil count and function [7, 8], and a decrease in the incidence of clinical mastitis [9, 10] compared to control cows. The objective of this study was to determine the effects of pegbovigrastim, a commercially available form of PEG-rbG-CSF, on complete blood counts in peripheral circulation when administered 7 days before anticipated calving and within 24 h after calving, according to the product label. In particular, we wanted to evaluate leukocyte counts at both the time of each injection and 24 h later and to characterize the response to each treatment. It was hypothesized that compared to control cows, treated cows would have elevated neutrophil counts following injections.

## Materials and methods

The study was designed as a double-blind, randomized controlled trial with two treatment groups; the present data are a subset from a larger clinical trial. Thirty-four primiparous (*n* = 15) and multiparous (*n* = 19) Holstein cows were enrolled in two high-producing commercial dairy herds in Southern Ontario, Canada. Farm A contributed 19 cows (*n* = 10 pegbovigrastim; *n* = 9 control) and Farm B, 15 cows (*n* = 8 pegbovigrastim; *n* = 7 control). All study cows were housed in free-stall facilities, fed total mixed rations for ad libitum intake to meet their nutrient requirements, had water available ad libitum, and were milked 3 times daily in a parlour. The study was approved by the Animal Care Committee of University of Guelph, Ontario, Canada (AUP # 3642). Cows were cared for in accordance to the Code of Practice for the Care and Handling of Dairy Cattle (2009) and the farms were licensed and inspected by provincial authorities for animal care every second year. Cows were enrolled weekly approximately 7 d before their expected calving date (272 to 279 d of gestation). Each cow was randomly assigned to receive a 2.7 mL subcutaneous injection of either sterile physiological saline (0.9% sodium chloride) or 15 mg of pegbovigrastim (Imrestor; Elanco, Ontario, Canada), according to the label directions. Randomization was done formally with lists for each farm, balancing treatment assignments in permuted blocks of 4 animals. Pre-labelled, pre-filled study syringes identified only with a number were refrigerated (2–8°C) until use. Therefore, both investigators and farmers were blinded to treatment assignments. All cows received 2 injections of their assigned treatment, the first administered one week before expected calving, and the second within 24 h after calving. Samples were taken d -7 and d -6 relative to expected parturition, then d 0, d +1, d +7, and d +21 relative to actual parturition. In accordance to the product label that states the first dose may be administered 4 to 10 days before the anticipated calving date, we only included cows for which the interval from the first injection to calving fell within this range. Thus, the interval (mean ± SD) from first treatment to calving was 6.7 ± 1.9 d. Approximately 10 mL of blood was collected from the coccygeal vessels into evacuated plastic tubes containing EDTA-K2. Whole blood samples were sent to the University of Guelph Animal Health Laboratory (AHL) within 2 h of collection and were analysed using an ADVIA 2120/2120i Hematology System machine differential (Siemens Healthcare Diagnostics Inc., Deerfield, IL). The initial machine differential did not provide the number of band versus segmented neutrophils. Therefore, to further describe the difference between the proportion of band neutrophils and segmented mature neutrophils, a single blood sample was taken 1 d after injection from 14 randomly selected cows. Each of these cows were treated with either saline or pegbovigrastim as described above, but were separate to the 34 cows detailed below. Samples were sent to the AHL within 1 h of collection and underwent an additional manual differential leukocyte assessment.

Data can be found in S1 Dataset and all statistical analyses were performed using SAS software, version 9.4 (SAS Institute Inc., Cary, NC, USA). Continuous outcomes were log_10_ transformed if distribution analysis and Shapiro-Wilk’s test determined that they were not normally distributed. The effect of pegbovigrastim on circulating blood counts was tested using multilevel mixed linear regression models. Farm was included as a random effect and time was considered a repeated effect, with individual cow as the model subject and an ante-dependence covariance structure. The following variables were tested: treatment, time, parity, body condition score (BCS) at enrollment, the interval between injections, and first-order interactions with treatment. A significance level of α = 0.05 was used. Manual backward step-wise elimination determined the fixed effects of the model. Because the main effects of treatment and time were important variables of interest for this study, they were forced into each model. Area under the curve (AUC) was calculated for each cow using the trapezoidal method [11] and a mixed linear regression model with farm as a random effect was constructed to compare the effect of treatment on neutrophil count AUC throughout the study.

## Results

**Fig 1:**
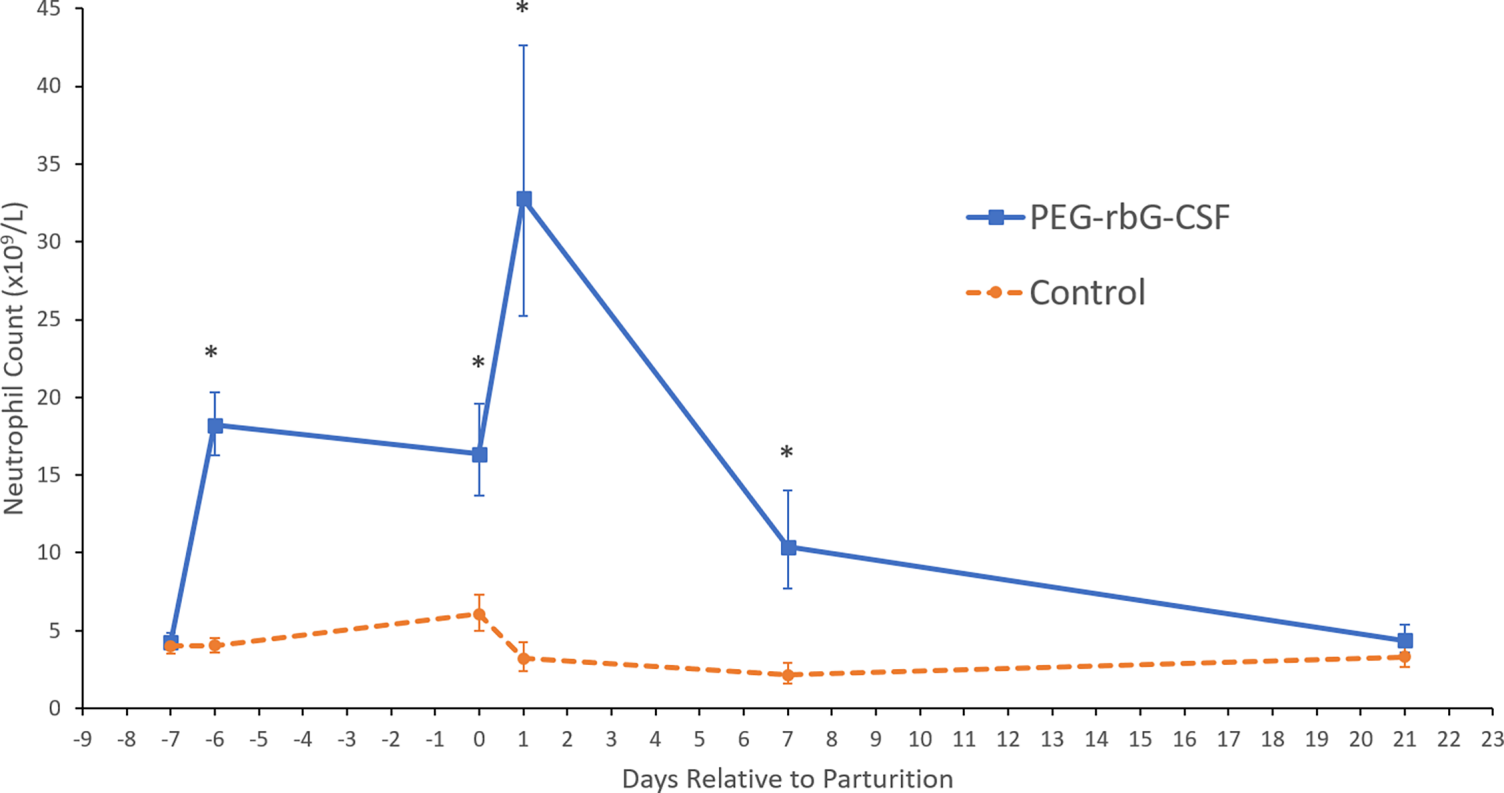
Effect of pegbovigrastim treatment on circulating neutrophil count in dairy cattle. Back-transformed least-squares means and 95% confidence intervals of circulating blood neutrophil count (×10^9^/L) for cows treated with 2 subcutaneous injections of pegbovigrastim (n = 18) (solid line) or saline (n = 16) (dashed line) at d -7 and d 0. Day -7 was relative to expected calving, with the actual mean interval = 6.7 ± 1.9 d. Asterisks indicate significant differences (*P* < 0.05) between pegbovigrastim and saline groups.

Neutrophil, monocyte, eosinophil, and basophil counts were not normally distributed, so were log_10_ transformed. Results are reported as back-transformed least-squares means, with 95% confidence intervals (CI). Detailed results are presented in Table 1. There were significant interactions between treatment and time for neutrophil, lymphocyte, and eosinophil counts. Treatment did not have a significant effect on red blood cell, monocyte, or basophil counts (*P* = 0.34, *P* = 0.61, and *P* = 0.94 respectively), whereas time and parity did. Pre-partum BCS and the interval between injections had no effect on any blood parameters and no interactions with treatment (*P* > 0.2). All hematology counts were relatively constant throughout the study for cows in the control group. Cows treated with pegbovigrastim had substantial, additive increases in circulating neutrophil counts after each injection. Neutrophil counts in pegbovigrastim-treated cows were significantly higher than in control cows on d -6, d 0, d +1 and d +7, (Fig 1**)** (*P* < 0.0001). After the first injection, neutrophil counts (×10^9^/ L) in pegbovigrastim-treated cows significantly increased from 4.3 (CI 3.8–4.8) at d -7 to 18.2 (CI 16.3–20.3) at d -6 (*P* < 0.0001). Their counts then decreased significantly from d -6 to d 0, when the second injection was administered, at a rate of -0.31 ×10^9^ neutrophils/L/day (*P* < 0.0001). After the second injection, neutrophil counts increased from 16.4 (CI 13.7–19.6) at d 0 to 32.8 (CI 25.2–42.7) at d +1 (*P* < 0.0001), after which counts decreased at a rate of -3.73 ×10^9^ neutrophils/L/day until d +7 (*P* < 0.0001). Counts continued to decrease from d +7 to d +21 at a slower rate of -0.43 ×10^9^ neutrophils/L/day (*P* < 0.0001), until baseline levels were reached. Conversely, in control cows, neutrophil counts were unchanged from d -7 to d -6 (*P* = 0.86) after the first injection and then decreased from 6.1 (CI 5.0–7.3) at d 0, to 3.2 (CI 2.4–4.2) at d +1 (*P* < 0.0001) after the second injection. The AUC (cells ×10^9^/ L per 28 d) for neutrophil counts in the pegbovigrastim-treated group was 429, versus 99 in the control group (*P* < 0.0001). In the separate group of cows with the manual differential leukocyte assessment, those in the control group (*n* = 5) had no band cells detected, with 100% segmented neutrophils. On average in the pegbovigrastim group (n = 9), band cells represented 5% (ranging from 1.3% to 9.6%) of the total neutrophil count, with the rest being segmented mature neutrophils.

**Table 1:** Effect of pegbovigrastim treatment on circulating leukocyte counts in periparturient dairy cattle at various sample times.

		Sample time (days from calving)						*P*- Value			
		-7	-6	0	1	7	21	Treatment	Time	Treatment × Time	Parity
Leukocyte Type	Treatment Group	Mean (95% Confidence Interval)									
Neutrophils	Control	4.01	4.05	6.06	3.20	2.15	3.29	<0.0001	<0.0001	<0.0001	0.0001
(× 10^9^ /L)	(*n* = 16)	(3.51–4.58)	(3.60–4.55)	(5.01–7.34)	(2.42–4.23)	(1.57–2.96)	(2.65–4.09)				
	Pegbovigrastim	4.28	18.21	16.36	32.80	10.39	4.37				
	(*n* = 18)	(3.77–4.84)	(16.30–20.33)	(13.66–19.58)	(25.22–42.66)	(7.70–14.02)	(3.56–5.36)				
	*P* value	0.46	<0.0001	<0.0001	<0.0001	<0.0001	0.05				
Lymphocytes	Control	2.97	2.68	2.45	2.61	2.73	2.63	0.04	<0.0001	0.04	<0.0001
(× 10^9^ /L)	(*n* = 16)	(2.16–3.78)	(1.81–3.54)	(1.61–3.29)	(1.73–3.50)	(1.90–3.56)	(1.79–3.47)				
	Pegbovigrastim	2.77	1.67	2.32	2.39	2.20	2.71				
	(*n* = 18)	(1.97–3.57)	(0.92–2.52)	(1.29–3.15)	(1.52–3.25)	(1.38–3.02)	(1.88–3.53)				
	*P* value	0.41	0.002	0.66	0.51	0.05	0.78				
Eosinophils	Control	0.25	0.25	0.16	0.17	0.12	0.2	0.0001	<0.0001	0.001	0.48
(× 10^9^ /L)	(*n* = 16)	(0.19–0.31)	(0.20–0.31)	(0.10–0.25)	(0.08–0.33)	(0.06–0.22)	(0.13–0.28)				
	Pegbovigrastim	0.33	0.3	0.12	0.07	0.06	0.2				
	(*n* = 18)	(0.27–0.41)	(0.25–0.38)	(0.07–0.18)	(0.04–0.14)	(0.04–0.11)	(0.14–0.28)				
	*P* value	0.06	0.19	0.3	0.08	0.15	0.93				

Back-transformed least-squares means (LSM) of blood leukocyte counts for cows treated with subcutaneous injections of pegbovigrastim or saline (control) 7 d before expected calving and within 24 h after calving. The P-value of the LSM difference between treatment groups is shown at each time point.

## Discussion

This study is the first to report neutrophil counts at both the time of injection and the response 24 h following each injection of pegbovigrastim. Cows that received pegbovigrastim had significant, marked increases in neutrophil counts of approximately 15 ×10^9^/L, 24 h after each injection. The increased neutrophil count was expected, because G-CSF is a growth factor related specifically to neutrophil differentiation [4]. Cows were able to sustain the elevated count after the first injection until calving. We show that there is another significant, additive elevation in neutrophil count on d +1, 24 h after the second injection. This increase was similar in magnitude to the increase at d -6 and resulted in an absolute count at d +1 approximately 10 times that of the control group. According to Paape et al. (2002), bovine neutrophils require 10 to 14 d to fully mature from a myeloblast to a mature neutrophil in bone marrow [12]. In marrow, maturation pools contain metamyelocytes and band cells, while storage pools contain band cells and mature neutrophils [13]. Naturally in a cow, neutrophils in storage pools appear in circulation after 7 d, where they have a half-life of about 9 h [12, 13]. The rapid increase in circulating neutrophil count following pegbovigrastim injection is hypothesized to result from the stimulated release of neutrophils from storage pools in bone marrow, containing both band cells and mature neutrophils. In treated cows, 5% of neutrophils in circulation were band cells, but no band cells were found in control cows, suggesting that pegbovigrastim stimulates the release of both immature and mature storage pools, but primarily the latter. The speed and magnitude of the observed increases in neutrophil counts indicate that there is a considerable reserve of mature and band cells in the bone marrow of transition dairy cows.

There is a substantial transient increase in circulating cortisol concentrations at parturition which causes down-regulation of expression of L-selectin on neutrophils, resulting in a release of marginated neutrophils to circulation [14], which would be expected to increase counts in circulation. However, consistent with other studies, in control cows, there was a slight decrease in neutrophil count immediately following parturition, consistent with mature neutrophils leaving circulation and migrating to sites of inflammation or infection, likely the uterus and udder [15]. The rates of decrease in circulating neutrophil counts after injection of pegbovigrastim were lower than would be expected based on the reported half-life of neutrophils [12], which is consistent with the combined effects of the treatment to provide ongoing stimulation of maturation and release, as well as to inhibit apoptosis of neutrophils [16].

Because the pegbovigrastim group had a significantly greater number of circulating mature neutrophils after parturition, cows may be better equipped to respond to pathogens, consistent with the lower incidence of clinical mastitis reported in clinical trials [9, 10]. At 7 d postpartum, there was still a significant difference between treatment groups, but not at 21 d. This provides the cow with an elevated immune capacity throughout the first 1 to 2 weeks of lactation when the risk of mastitis and reproductive disease is high. Our results regarding neutrophil count are consistent with previous studies. Others reported immediate increases in neutrophil count following the first injection [7, 9, 10] or second injection [6], similar in magnitude to those found in the current study. However, until now these responses have not been compared to the neutrophil count at the time of injections. Kimura et al. [6] also analyzed band cell counts and found very few band cells present in control cows, and a significantly elevated number of band cells in treated cows, especially in response to the second injection. Our results suggest that the responses to pegbovigrastim do not deplete storage pools of mature neutrophils because the response to the second injection was of the same absolute magnitude as the first, and the great majority of these cells were segmented neutrophils.

Basophils, eosinophils, and monocytes, like neutrophils, are all derived from myeloblasts, but their differentiation is directed by growth factors other than G-CSF. The lack of effect of treatment on basophil and monocyte counts shows that pegbovigrastim does not influence their circulating levels. Eosinophil counts were affected by treatment, but the magnitude of effect was minute and the differences in counts were not considered biologically important. Lymphocytes are derived from lymphoid progenitors and should therefore not be affected by G-CSF. However, treatment did have a significant effect on lymphocyte count at d -6, although counts were broadly stable over time for both treatment groups, and it is unclear if this transient difference is of biological importance. In contrast to this result, McDougall et al. (2017), reported a substantial response to treatment in both monocyte and lymphocyte counts [7].

## Conclusions

We conclude that injection of pegbovigrastim triggers a prompt and sustained increase in circulating neutrophil count in periparturient dairy cows. The injection of pegbovigrastim one week prior to the expected calving date triggers a rapid increase in neutrophil count. There was an additive increase following the second injection. The contribution of approximately 5% band cells to the responses in pegbovigrastim-treated cows demonstrates that this rapid increase is most likely a release from bone marrow storage pools instead of maturative pools, but that the storage pool reserve is not depleted by pegbovigrastim. We encourage additional large-scale randomized controlled trials to confirm the effects of pegbovigrastim on the risk of reproductive and infectious disease in dairy cows during the transition period.

## Supporting information

S1 Dataset Dataset for the effects of pegbovigrastim on circulating leukocyte counts in periparturient dairy cattle. (CSV)
